# Supplementary material for: Enhanced Algal Photosynthetic Photon Efficiency by Pulsed Light
Source: iScience. 2020 Apr 29;23(5):101115. doi: 10.1016/j.isci.2020.101115 (PMC7235644; doi:10.1016/j.isci.2020.101115)
Supplement: Document S1. Transparent methods, Figures S1–S6, and Tables S1 and S2 [file mmc1.pdf]

iScience, Volume *23*

Supplemental Information

Enhanced Algal Photosynthetic Photon Efficiency by Pulsed Light

Yair Zarmi, Jeffrey M. Gordon, Amit Mahulkar, Avinash R. Khopkar, Smita D. Patil, Arun Banerjee, Badari Gade Reddy, Thomas P. Griffin, and Ajit Sapre

Supplemental Information

Results when N_{pool} and A can vary

For the case with T_{pulse} = 150 ms and T_{dark} = 250 ms (Fig. S1a), the predicted trend is opposite to that of the data. Whereas the measured η_{ph} decreases as I is increased, the model predicts an increase in η_{ph} (Eq. (11)). The predicted increase is predicated on both A and N_{pool} having constant values. However, photo-acclimation can cause a reduction in A and N_{pool} as I is increased (de Wijn and van Gorkom, 2001; Zou and Richmond, 2000; Simionato et al., 2011; Bonente et al., 2012; Gris et al., 2014). Figure S1b illustrates that the correct trend can be attained if it is assumed that photo-acclimation induces changes in A and N_{Pool} as I is increased (Table S1 lists values that are reasonable based on prior measurements, but do not signify actual observed parameters).

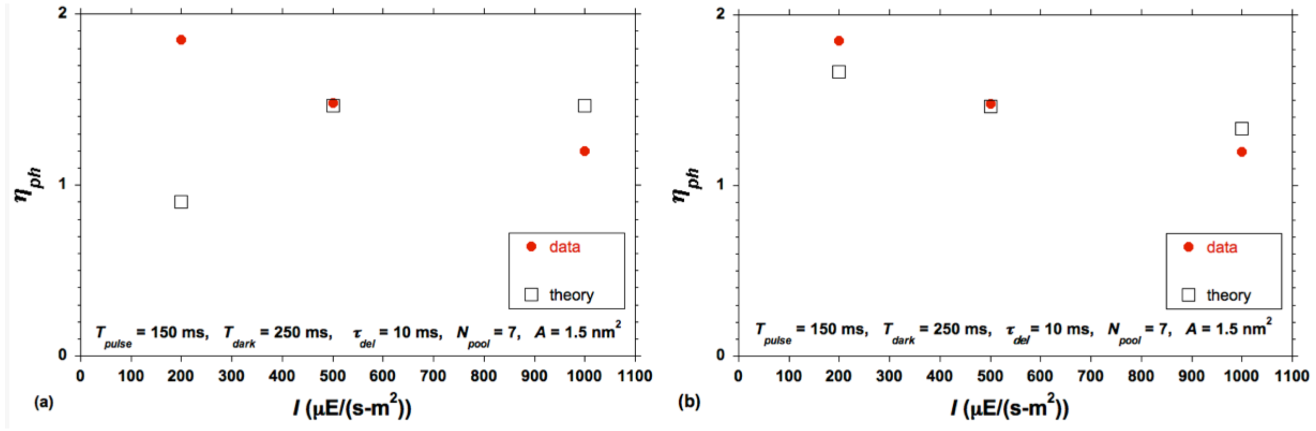

Fig. S1. Related to Fig. 6c. (a) Measured and calculated (Eq. (11)) η_{ph} vs. I for T_{pulse} = 150 ms, T_{dark} = 250 ms (same as Fig. 6c where the standard deviations and number of replications for the measured points are noted). (b) Modification of model predictions of part (a) when model parameters vary with I owing to photo-acclimation as in Table S1. The data points are the same as in part (a).

Figure S2a shows model predictions when a distribution of N_{pool} is accounted for (Eq. (17)), with average $<N_{pool}>$ = 7 and standard deviation σ = 2. Figure S2b shows the same data but with model calculations that allow for the effect of photo-acclimation on A and N_{pool} as listed in Table S2. The effect of photon arrival time statistics has been incorporated.

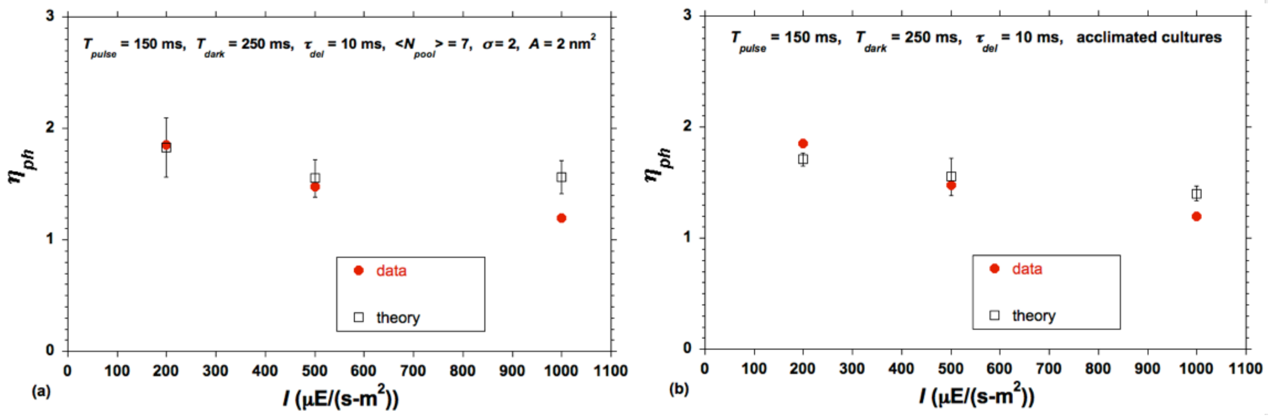

Fig. S2. Related to Fig. 6c. Dependence of η_{ph} on I at the relatively long T_{pulse} = 150 ms. The measured data and their standard deviations are the same as in Fig. S1. Model predictions account for (i) a distribution of N_{pool}, and (ii) the possible impact of photo-acclimation on A and N_{pool}. Vertical bars for the model (computed) results correspond to the inherent standard deviations associated with photon-arrival statistics, as elaborated in the text.

Figure S3 further sharpens this point with a comparison between data from (Simionato et al., 2013) and model predictions when a distribution of N_{pool} is accounted for. In all these computations with the full statistical model, each reaction center was randomly assigned a value of N_{pool} using the probability density of Eq. (17).

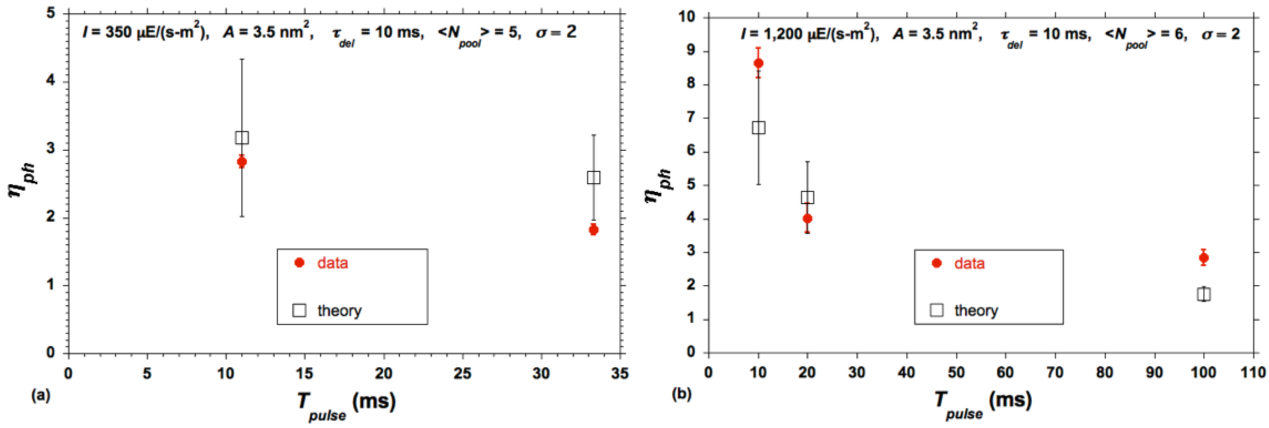

Fig. S3. Related to Fig. 7. Comparison of data from (Simionato et al., 2013) against model predictions for the dependence of η_{ph} on T_{pulse} at (a) low and (b) intermediate I values. The theory accounts for a distribution of N_{pool}. The vertical bars of ±1 standard deviation about the average: (i) were taken from (Simionato et al., 2013) for the measured data, for 3 replications, and (ii) correspond to the inherent standard deviations associated with photon-arrival statistics for the model (computed) results, as elaborated in the text.

Table S1. Related to Fig. 6c. Parameter values used for the model predictions in Fig. S1, based on the possible impact of photo-acclimation.

I (μE/(m^2-s)	A (nm^2)	N_{Pool}
200	4	10
500	2	7
1000	1	5

Table S2. Related to Fig. 6c. Parameter values for model calculations in Fig. S2, accounting for the possible effect of photo-acclimation.

I (μE/(m^2-s))	A (nm^2)	N_{Pool}
200	4	9
500	2	7
1000	1	5

Full statistical analysis

Sample distributions are presented in Fig. S4 for I = 1000 μE/(m^2-s), A = 1 nm^2, T_{pulse} = 10 ms, τ_{del} = 10 ms and N_{Pool} = 7. Figure S4a reflects the fact that the number of photons absorbed during the pulse is small. The average number of photons hitting A = 1 nm^2 during a 10 ms pulse is 6.02. The actual number varies randomly from one reaction center to another, depending on the randomly varying time gaps between photons. Hence, the probability of fully reducing the PQ pool is negligible, as is the probability of losing a large number of photons.

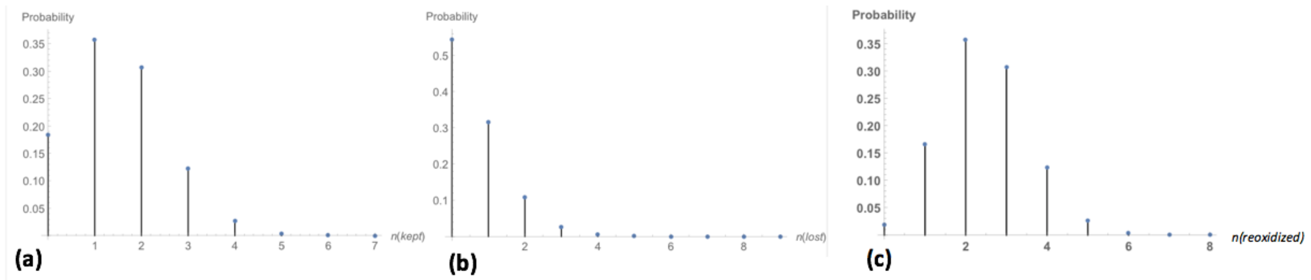

Fig. S4. Related to Fig. 8. Probability distribution for the number of (a) PQs stored in the pool, (b) photons lost by the end of a 10 ms pulse, (c) PQs reduced over a cycle with T_{pulse} = 10 ms and a sufficiently long dark time.

The situation is quite different for long pulses. Examples are presented in Figs. S5 and S6 for I = 1,000 μE/(m^2-s), A = 1 nm^2, τ_{del} = 10 ms and N_{Pool} = 7, but with a longer T_{pulse} = 150 ms. Owing to the long pulse time, the probability that the PQ pool is completely reduced at the end of the pulse is high (Fig. S5a). The high number of lost photons (Fig. S6) is a consequence of the fact that the PQ pool is fully reduced shortly after the start of the pulse, so that many photons are lost due to reduced charge carriers.

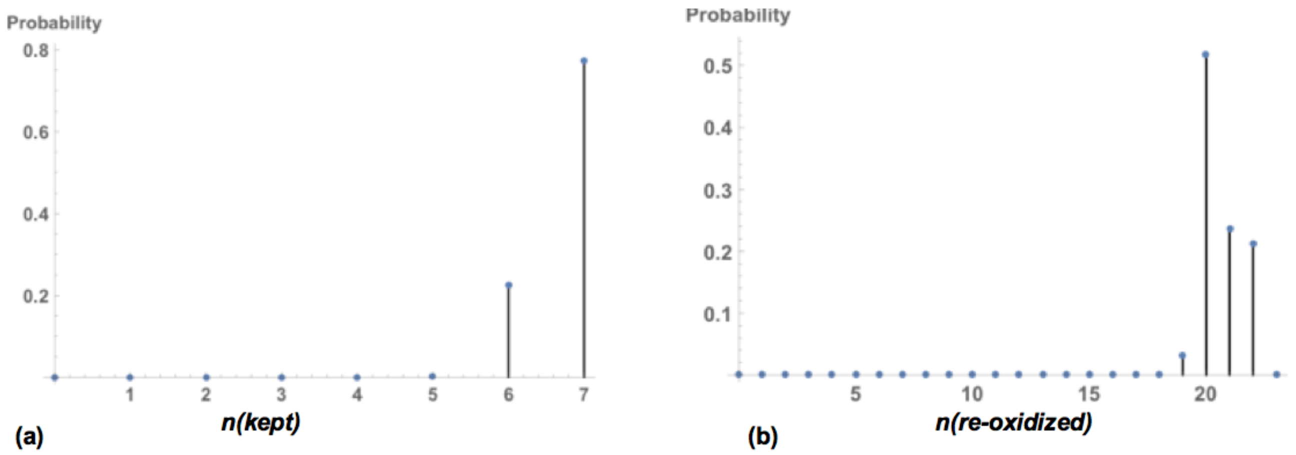

Fig. S5. Related to Fig. 6c. Probability distribution for (a) the number of PQs reduced in the pool at the end of a 150 ms pulse and (b) the number of PQs re-oxidized by the end of a cycle with T_{pulse} = 150 ms and a sufficiently long dark time.

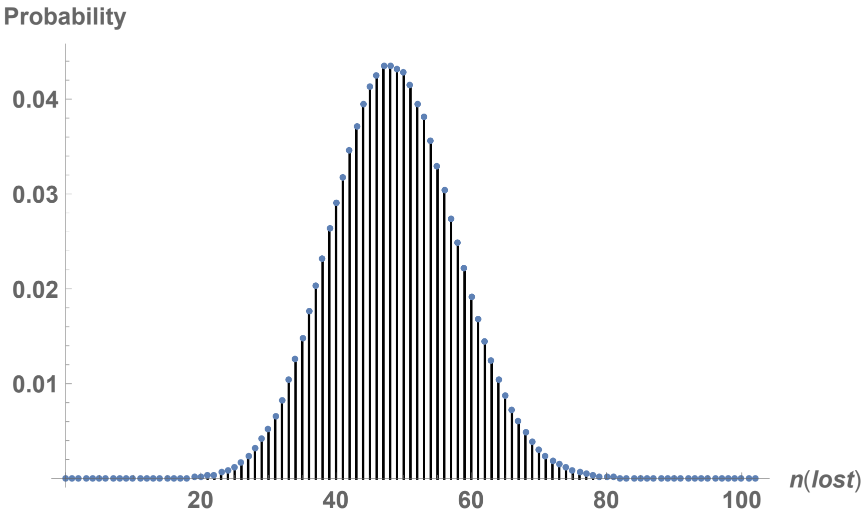

Fig. S6. Related to Fig. 6c. Probability distribution for the number of photons lost during a 150 ms pulse.

Using the approach of the simple average model, Eq. (5) yields the number of reduced PQs to be 45. Of these, 15 are re-oxidized during the pulse (Eq. (7)), from which 7 PQs can stay reduced. Hence, 22 PQs can be re-oxidized during one cycle. The statistics of photon arrival times, combined with the losses owing to the 0.2

ms time scale, reduce this number to an average of 20.63, in which case η_{ph} = 20.63/($T_{pulse} \cdot \tau_{del}$) = 20.63/(150·10) = 1.375.

Transparent Methods

We used a locally isolated strain of the *Nanochloris* species (which is a green microalgae) from our repository, cultivated in urea-phosphoric acid medium (urea 214 ppm and phosphoric acid 31 ppm, prepared in artificial seawater 4% by weight) and maintained at 27°C and 300 μE/(m^2-s), at an optical density of 2 in a 500 ml flask.

Biomass growth curves were generated using a Multi-Cultivator MC 1000-OD of Photon Systems Instruments (PSI, Czech Republic), comprising 8 test-tubes, each holding 70 ml of algal culture, and immersed in a water bath maintained at 35°C. Dilute algal cultures were used (density 17-30 mg/l, i.e., 0.05 OD, < 10% light attenuation), to ensure that all cells experienced essentially the same light intensity. pH was maintained at 7.0 by sparging humidifed air with 2% CO_2 at an air flow rate of 1 VVM (70 ml/min). Each test tube was irradiated by its own cool-white LED array.

For pulsed-light experiments, four PSI light sources (Model SL-3500, with an LC-100 PSI light controller) permitted independently tuning the irradiation and dark times from 1 ms to 999 ms. Our small glass reactors had optical path 3 cm, width 10 cm and height 15 cm. An operating height of 10 cm was used, so the total fluid volume was 300 ml.

For both continuous and pulsed irradiation, LEDs were controlled such that the instantaneous photon flux density for photosynthetically-active radiation at the surface of the culture was 1000 μE/(m^2-s), measured using Apogee Instruments' quantum meter model MQ-200. The 13 cm × 13 cm LED panel was sited less than 1 cm from the reactor. Light intensity was measured at the center of each of 9 equal-area regions comprising the reactor's illuminated surface, and the reported I = 1,000 μE/(m^2-s) represents the average over these 9 sections.

Growth was measured over an illumination period of 6 hours, followed by a period of 6 hours of dark time, after which a fresh run was started, with these cycles repeated for 24 hours. Each day, the culture was harvested and brought to the desired starting operating optical density of 0.08, measured at a wavelength of 750 nm at the start (OD_1) and end of irradiation (OD_2) to get specific growth rate μ over time t: $\mu = \frac{ln(OD_2/OD_1)}{t}$. All runs were repeated ~20 times, from which average and standard deviation values were determined.
